# Turner Syndrome Mosaicism 45,X/46,XY with Genital Ambiguity and Duchenne Muscular Dystrophy: Translational Approach of a Rare Italian Case

**DOI:** 10.3390/ijms232214408

**Published:** 2022-11-19

**Authors:** Bruno Lamanna, Marina Vinciguerra, Miriam Dellino, Gabriele Cascella, Gerardo Cazzato, Enrica Macorano, Antonio Malvasi, Salvatore Scacco, Ettore Cicinelli, Vera Loizzi, Antonella Vimercati, Gennaro Cormio, Francesco Paduano, Eliano Cascardi, Marco Tatullo

**Affiliations:** 1 Department of Biomedical Sciences and Human Oncology, University of Bari “Aldo Moro”, 70121 Bari, Italy; 2 Fetal Medicine Research Institute, King’s College Hospital, London SE5 9RS, UK; 3 Department of Emergency and Organ Transplantation, University of Bari “Aldo Moro”, 70121 Bari, Italy; 4 Section of Legal Medicine, Interdisciplinary Department of Medicine, University of Bari “Aldo Moro”, 70121 Bari, Italy; 5 Department of Translational Biomedicine and Neuroscience (DiBraiN), University of Bari “Aldo Moro”, 70121 Bari, Italy; 6 Gynecologic Oncology Unit, IRCCS Istituto Tumori Giovanni Paolo II, Department of Interdisciplinary Medicine (DIM), University of Bari “Aldo Moro”, 70121 Bari, Italy; 7 Stem Cells and Medical Genetics Units, Tecnologica Research Institute and Marrelli Health, 88900 Crotone, Italy; 8 Department of Medical Sciences, University of Turin, 10124 Turin, Italy; 9 Pathology Unit, FPO-IRCCS Candiolo Cancer Institute, 10060 Candiolo, Italy; 10 Honorary Senior Clinical Lecturer—University of Dundee, Dundee DD1 4HR, UK; 11 Founding Member of MIRROR—Medical Institute for Regeneration and Repairing and Organ Replacement, Interdepartmental Center, University of Bari “Aldo Moro”, 70124 Bari, Italy

**Keywords:** Turner syndrome (TS), Duchenne muscular dystrophy (DMD), translational medicine, ambiguous genitalia

## Abstract

Turner syndrome (gonadal dysgenesis with short stature and sterility) is characterized by chromosomal karyotype 45,X in 50% of cases or by mosaicism (45,X/46,XX and 45,X/46,XY) in 30–40% or X structural defects (deletions, long arm isochromosome, ring chromosome). When mosaic Turner syndrome (TS) occurs with a Y chromosome, there may be ambiguous genitalia. Duchenne muscular dystrophy (DMD) is an inherited neuromuscular disease with an X-Linked recessive pattern of inheritance that predominantly affects males, while females are usually asymptomatic. DMD has also been observed in groups of females affected by TS, not homozygous for the mutation. Here, we report a case of an Indian neonate born with ambiguous genitalia diagnosed prenatally by ultrasound who had a karyotype of 45,X/46,XY and who also had Duchenne muscular dystrophy caused by a de novo mutation in the DMD gene. Physical examination was normal without the typical dysmorphic features of TS with the exception of the genitourinary system showing ambiguous genitalia. Gender was assigned as female. At the age of three years, she had increasing difficulty walking, running, jumping and climbing stairs, proximal upper and lower extremity muscle weakness and a positive Gowers’ sign. In addition, the serum creatine kinase (CK) value was over 30X the upper limit of normal. This study shows that DMD can occur in females with TS having 45,X/46,XY mosaicism and that this coexistence should be considered in women affected by TS who start to develop potential typical symptoms such as motor or developmental delay.

## 1. Introduction

Many factors contribute to sexual development. The four key factors are (i) the chromosome of the zygote (46,XX in females; 46,XY in males), (ii) the differentiation of the gonads into the ovary and testis, (iii) the differentiation of the organs responsible for reproduction and development of the external genitalia, (iv) normal function of adrenal steroid synthesis and metabolism (in the adrenal cortex and liver, respectively). The sex chromosome Y provides the signal for the development of the male gonad, regardless of the number of female sex chromosomes (X chromosome) present [1]. The absence of the Y orients the development of the individual in a female direction. From the genetic sex which is formed at the moment of conception in the chromosomal structure (46,XX or 46,XY) follows a series of modifications which lead to the formation of the female (ovary) or male (testicular) gonad and thus to the definition of the gonadal sex of the person [2]. The gonads secrete hormones that control the development of the external genitalia (phenotypic sex). Sexual differentiation begins in the human embryo only after the 6th week [3,4]. The phenotypic sex of the embryo, therefore, depends on the determination of the gonadal sex which is primarily related to the complement of the sex chromosomes and the presence/absence of the Y chromosome. However, very few exceptions are known; testicular feminisation syndrome can account for phenotypic females who have an XY karyotype and is usually due to mutations in the X-linked androgen receptor gene; XX male syndrome, also known as de la Chapelle syndrome, is a rare congenital intersex condition in which an individual with a 46,XX karyotype has phenotypically male characteristics [5].

In humans, the Y chromosome determines the male sex. When there is only one X chromosome (Karyotype 45,X or monosomy X), the phenotype is female and corresponds to Turner syndrome (TS), which is characterized by gonadic dysgenesis, short stature and sterility [6]. Conversely, the sex chromosome complement XXY (47,XXY or Klinefelter syndrome) and its more complex variants (48,XXXY; 49,XXXXY) give rise to a predominantly sterile male phenotype [7]. However, if there is somatic cell mosaicism (coexistence of genetically different cell lines) 45,X/46XX or 45,X/46,XY, the phenotype varies from a sterile male (when the XY line prevails) to Turner syndrome (ovarian dysgenesis with short stature, when the 45,X line X prevails), with intermediate cases of dysgenesis of the gonads and ambiguity of the genitals. Turner syndrome is characterized by chromosomal karyotype 45,X (monosomy X, also called 45,XO) in 50% of cases, or by mosaicism in 30–40%, most commonly 45,X/46,XX and less commonly 45,X/46,XY, or by X structural defects (deletions, long arm isochromosome, ring chromosome) [8]. People affected by the syndrome are phenotypically female, with dysgenetic ovaries, hypoplasia of the uterus and tubes, do not have pubertal maturation or menarche, do not produce female gametes and show hypoplasia of secondary sexual characteristics. The external genitals retain a childlike appearance, the stature is short, the chest is deformed; the intellectual deficit is rarely present. The forms of Turner syndrome with mosaicism, having more than one cell line, show heterogeneity in phenotypic manifestations such as alterations of the gonadal tissue, ambiguity of the external genitalia with pubertal delay, and primary amenorrhea in people with a female phenotype [9]. Turner syndrome occurs in 1 of every 2000–2500 live female births [10]. 

Patients with X-linked recessive disease are typically male and have mutations in genes on their X-chromosome. This chromosome is inherited from their mother, and the altered gene can be carried by her, usually asymptomatically, or arise from de novo mutation. Fully affected females are rare, either being homozygous through having mutations in both copies of their X-linked gene (one of which will be the copy from their father, who may himself have the same condition) [11], or through having only a single intact X-chromosome (as in Turner syndrome), and which happens to carry a mutation in the relevant gene. However, in several X-linked “recessive” disorders, patients’ mothers can have some clinical evidence of the condition because of functional mosaicism [12].

Duchenne muscular dystrophy (DMD) is an inherited neuromuscular disease with an X-Linked recessive pattern of inheritance that predominantly affects males with a prevalence of 1:3.500–5000 live male births [13,14], while females are usually asymptomatic (called healthy carriers). Duchenne muscular dystrophy and Becker muscular dystrophy (BMD) occur, respectively, due to a total or partial deficiency of a protein called dystrophin encoded by the *DMD* gene (dystrophin gene) located on the X chromosome (locus Xp21) [15]. Disease-causing mutations in the *DMD* gene typically involve deletion or duplication of exons (70% of cases) or sequence alterations (30%). DMD manifests itself in early childhood with problems in walking that progress to the loss of autonomy. The first symptoms of the disease manifest themselves at around two to three years of age. The child has trouble running, climbing stairs, and jumping. The weakness of the hip muscles causes the child to show the “Gowers’ sign”, a particular way of using the hands resting on the thighs to get up from the ground or a sitting position. With age, motor difficulties become obvious and worsen together with deterioration in respiratory and heart function. DMD has also been observed in groups of females affected by Turner syndrome. Based on the incidence of the two disorders, the coexistence of TS and DMD may occur with a frequency of 1.4 × 10^−7^ [16].

## 2. Case Presentation

**Figure 1:**
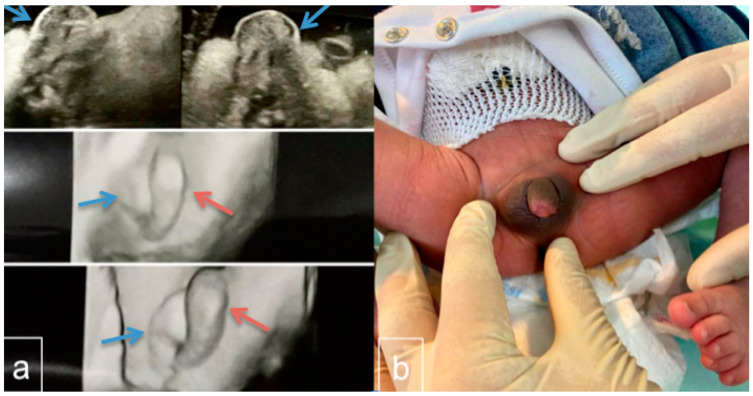
(**a**) Ultrasound scan, 2D and 3D, of the fetal genitalia done in the second trimester. Visualization of the vulva, clitoris, and labia is considered to indicate a female fetus whereas demonstration of the scrotum, penis, descended testicles, and penile midline raphe is interpreted as indicative of a male. In the ultrasound image, there is a labial/scrotum-like formation (blue arrow) and a median prominence similar to the phallus/clitoris (red arrow). (**b**) Infant 24 h after birth with ambiguous genitalia. The phallus was 1.4 cm in length with a blind dimple on the glans and a urethra that opened ventrally at the base, consistent with Prader 4 hypospadias. The clitoral index was 0.7. The anogenital ratio was 0.78. No gonads were palpable in the labial/scrotal tissue. Pelvic imaging did not identify a uterus or ovary, but only small formations in the inguinal duct.

We report a case of a child diagnosed prenatally (by ultrasound) as having genital ambiguity after referral to our unit of Obstetrics and Gynecology. An Italian neonate of Indian heritage was born at term via cesarean section (breech presentation) with a birth weight of 3.330 kg and height of 48 cm. The mother was 27 and the father was 28 years old. Family history was negative for consanguinity or infantile deaths. First-trimester serum screening revealed a low risk of trisomy. However, a fetus with ambiguous genitalia was identified at the second-trimester scan (Figure 1a). No other anomalies were found with regular fetal growth. An amniocentesis was offered but kindly declined. The fetus was active, and there were no complications at birth (Apgar of 9–10 at both five and ten minutes). 

At birth, physical examination of the infant reported normal results without the typical dysmorphic features of TS except for the genitourinary system which showed ambiguous genitalia. The phallus was 1.4 cm in length with a blind dimple on the glans and a urethra that opened ventrally at the base, consistent with Prader 4 hypospadias [17]. The clitoral index was 0.7. whereas the anogenital ratio was 0.78. No gonads were palpable in the labial/scrotal tissue (Figure 1b). At birth, a transabdominal ultrasound was performed on the neonate, which did not show any evidence of the presence of the uterus and ovaries, but only small formations in the inguinal duct, and could not distinguish whether the uterus and ovaries were unusually small or altogether absent. Thus, the mother was offered an MRI examination on her baby, which was refused. Echocardiography and renal sonography revealed no abnormalities. A standard chromosome analysis based on 10 metaphases from cultivated peripheral lymphocytes with Q-banding showed a karyotype of 45,X/46,XY mosaicism. Here, 7 out of 10 metaphases contained a 45,X cell line while the remaining 3 contained 46,XY. The infant was assigned a female gender.

This mosaic chromosome result was confirmed by a second karyotype, using fluorescence in situ hybridization (FISH) analysis, in peripheral lymphocytes screening 100 metaphase lymphocytes at 400–450 band resolution with specific probes for chromosome X. Out of 100 metaphases, 89 presented 45,X karyotype, while the remaining 11 presented regular 46,XY karyotype. Due to the presence of a Y chromosome, after careful discussion with the urologist and parents, a prophylactic gonadectomy was performed. Gonad histology showed a combination of testicular and ovarian tissue. Histological FISH analyses of the gonads revealed 75% of the cells had the Y chromosome, suggesting that the patient’s Karyotype was 45,X/46,XY with important discordance compared to the analysis on peripheral lymphocytes. 

During the first two years of life, the mother noticed a delay in the acquisition of motor stages in her daughter, which, at an initial consultation with the pediatrician, received no additional attention. At the age of three years, it was noted that she had increased difficulty walking, running, jumping and climbing stairs, proximal upper and lower extremity muscle weakness and a positive Gowers’ sign. Serum creatine kinase (CK) was 4567 IU/L (normal is 26/140 IU/L). Liver function and thyroid function tests produced results within normal limits. With the suspicion of a diagnosis of DMD, the DMD gene was first evaluated by MLPA (Multiplex Ligation-dependent Probe amplification) analysis to search for deletion/duplication, followed by Sanger sequencing of all the coding exons of the gene. A deletion of exons 46–49 in the *DMD* gene was observed (which is predicted to affect the reading frame), confirming the diagnosis [18]. 

Electromyography and muscle biopsy also confirmed the diagnosis [19]. A biopsy from the left quadriceps muscle showed an abnormal number of hyaline fibres, necrotic fibres undergoing phagocytosis and chains of nuclei in regenerating fibres. Immunohistochemical analysis showed a total absence of dystrophin, therefore diagnostic of DMD [19]. 

Testing DNA from the mother was negative for the exon 46–49 deletion, indicating that the mutation in her daughter was de novo, although the risk of recurrence in future sons due to a potential for germinal mosaicism in the mother cannot be excluded. 

## 3. Discussion

Turner syndrome is a chromosomal syndrome characterized by short stature, gonadal dysgenesis (usually without ambiguity of the external genitalia, but with developmental defects of secondary sexual characteristics and infertility), characteristic signs of the external phenotype and anomalies of some internal organs [6]. In Turner syndrome, 50% of patients have a 45,X karyotype with no mosaicism and no ambiguity of the genitalia, 30–40% of cases have mostly 45,X/46,XX or 45,X/46,XY mosaicism. Patients with a Y chromosome may have ambiguous genitalia. The fact that these patients have more than one cell line may explain the variability of the phenotype in individuals with Turner syndrome. The literature reports that the percentage of the Y cell line in peripheral blood remains an unreliable indicator of sexual phenotypes [20,21]. In these articles, the authors compared the ratio of cell lines in different tissues of Turner syndrome patients, including peripheral blood lymphocytes, oral mucous cells, cutaneous fibroblasts, and gonadal tissue. They found that the cell ratio between cells showing 45,X monosomy and cells with an abnormal Y chromosome may differ from one tissue to another. These studies suggest that the determination and differentiation of the gonads of mosaic subjects mainly depend on the predominant cell line in the gonads. Importantly, 45,X leads to the development of striated gonads, while 46,X,idic(Y) or 46,XY leads to a dysgenetic testicle [22]. These results are consistent with the difference between peripheral blood karyotype, gonosome karyotype in gonadal tissue, and the sexual phenotype we observed in our case.

The importance of detecting the presence of Y chromosome material is that it confers an increased risk of benign and malignant germ cell tumors and prophylactic bilateral gonadectomy is recommended. Gonadoblastoma and dysgerminoma have been reported in girls with Turner mosaic who carry Y chromosome material. Prophylactic gonadectomy should be considered in these girls without delay [23]. Canto et al. examined the presence of Y-chromosome sequences in 107 Turner syndrome patients with a 45,X karyotype; this was found in 10 out of 107 cases (9.3%), of which 2 out of 10 cases (20%) were confirmed to have gonadoblastoma following prophylactic gonadectomy [24].

Like typical 46,XY male, a female with Turner syndrome also has an increased risk compared with typical 46,XX females of any X-linked recessive disorder; for example, Panarello et al. presented a concomitant case of Tuner syndrome and hemophilia A [25].

Consequently, the coexistence of an X-linked recessive disorder should be considered in women affected by Turner syndrome 45,X or with 45,X/46,XX or 45,X/46,XY mosaicism. Several cases are evident which indicate the coexistence of 45,X Turner syndrome and Duchenne muscular dystrophy. Ferrier et al. in 1965 and Wu et al. in 2019 described individual girls with mosaic 45,X/46,XX and 46,X,i(Xq) karyotype, respectively, who presented a coincidence of TS and DMD [26,27]. Ou et al. in 2010 and Bjerglund et al. in 1984 described individual female patients with DMD who carried an atypical karyotype of Turner syndrome with a structurally altered X chromosome and harbored a de novo mutation in the dystrophin gene in the remaining X chromosome [22,28]. A girl with normal genitalia that fully expressed DMD disease because of her 46,XY karyotype was reported by Wulfsberg in 1986 [29]. Kaczorowska et al. in 2016 described a 4½-year-old female with classical 45,X Turner syndrome who also had Duchenne muscular dystrophy caused by a point mutation in the *DMD* gene (c.9055delG) [16].

As mentioned, DMD is a recessive myopathy linked to the X chromosome and related to dystrophin deficiency in cardiac and skeletal muscles due to deletion/mutation of the *DMD* gene (Xp21.2). Females with clinical signs of DMD are usually carriers of X chromosome rearrangements or have non-random inactivation of the X chromosome or are affected by Turner syndrome (complete or partial absence of the X chromosome) (Table 1). 

This case raises the question of whether ambiguous genitalia recognised prenatally or at birth could be an indicator of a potential increased risk of DMD, in either males or females. De novo mutations are common in DMD and BMD; indeed, both of them are caused by de novo germline mutations in one-third of patients [37]. 

Most cases of ambiguous genitalia and subsequent DMD are coincidental occurrences. In rare cases, a de novo deletion in Xp21 may involve not only the DMD gene but also the adjacent *NR0B1* gene at Xp21.2, resulting in congenital adrenal hypoplasia with gonadotropin deficiency (though not usually ambiguous genitalia), as well as subsequent DMD arising from the same genetic mutational event. Similarly, it would be theoretically possible for a pericentric inversion on the X-chromosome to affect simultaneously both the Androgen receptor (*AR*) gene at Xq12 (causing androgen insensitivity/testicular feminisation) and the *DMD* gene at Xp21, but such cases would be exceptionally rare. 

Otherwise, the occurrence of DMD with other genetic causes of ambiguous genitalia with XY karyotype (e.g., partial androgen insensitivity from AR gene mutation) will be coincidental and occur at an incidence equal to the birth incidence of one multiplied by the birth incidence of the other. Similarly, in Turner syndrome or its variant and XY mosaic forms, the occurrence of a DMD mutation will be coincidental and have the same frequency of occurrence as seen with normal XY male births.

Mothers who are not somatic carriers of DMD mutations but who have children with DMD or BMD are at risk of having another child with DMD or BMD owing to germline mosaicism (a percentage of her oocytes carries the mutation). The frequency of germline mosaicism in oocytes or sperms varies per individual but can be up to 14% [38]. The presence of case literature that reports the coexistence of Turner syndrome and DMD leads us to consider the genetic investigations that should be conducted at the time of prenatal diagnosis of Turner syndrome. What should be the course of action of the gynecologist and geneticist facing the prenatal diagnosis of classical or mosaic Turner syndrome towards the patient? 

As in our case, and as the available literature shows, if a fetal syndrome 45,X or 45,X/46,XX or 45,X/46,XY mosaicism is suspected or confirmed by fetal DNA/amniocentesis, careful parental counselling should be offered about TS, considering not only the potential risk for gonadoblastoma, where a Y chromosome is present, but also the family history, and advising that their baby girl will have the same risk for X-linked disorders (such as haemophilia or DMD) as would any normal male infant of theirs, but equally that there is no increased risk where there is no previous known family history.

Communication of the diagnosis of a genetic disease, especially in association with another, always needs to be accompanied by careful genetic counselling; this is essential for providing information on the knowledge of possible nature of the disease, its risk of hereditary transmission, its natural history and possible medical interventions for its prevention and treatment. In Turner syndrome, the use of growth hormone (GH) alone or in combination with oxandrolone (OX) can stimulate growth in the short term and increase adult size in girls with Turner syndrome [39]. 

## 4. Conclusions

Turner syndrome occurs due to the complete or partial absence of one of the two X chromosomes and this defect may affect all the individual’s cells or only a percentage of them. The clinical picture is very varied and has a different genotypic–phenotypic expression depending on whether it is a monosomy X or mosaic form. The diagnosis is formulated on examination of leukocytes through a cytogenetic investigation but sometimes it is necessary to use cells from other tissues that in some cases may be discordant. The presence of a karyotype containing Y material (which should always be suspected in cases with ambiguous genitalia) should lead to gonadectomy due to an increased risk of gonadoblastoma degeneration. 

It is important to note that girls with a single intact X-chromosome as in Turner syndrome (45,X0) or mosaic forms have the same coincidental risk as normal male infants for X-linked recessive disorders such as DMD and that these should be considered if the girl develops any relevant symptoms of concern. The girl should also be included in any routine newborn or later childhood screening programme for those disorders if any of these were to be proposed. 

## Figures and Tables

**Table 1:** All cases of Turner and Duchenne syndrome which have been documented in the literature since the 1960s.

Paper ID	Year of Publication	Karyotype	Turner Phenotype	DMD Onset	DMD Symptoms	CPK at Onset (UI/L)	Origin	Mutation *DMD* Gene
Chen et al. [30]	2020	45,X	yes	9 yrs old	typical	6.566	China	Familiar
Verma et al. [31]	2017	45,X	yes	5 yrs old	typical	4504	USA	De novo
Kaczorowska et al. [16]	2016	45,X	yes	14 months old	atypical	20.451	Poland	-
Wu et al. [27]	2019	45,X	yes	8 yrs old	typical	-	China	-
Bjerglund et al. [22]	1984	45,X	yes	-	typical	-	Denmark	De novo
Chelly et al. [32]	1986	45,X	yes	2 yrs old	typical	-	France	De novo
Ou et al. [28]	2010	46,X,i(X)(q10)	no	4 yrs old	typical	-	China	De novo
Kinoshita et al. [33]	1990	45,X/46,XX/47,XXX	incomplete	52 yrs old	atypical	-	Japan	-
Wulfsberg et al. [29]	1986	46,XY	no	2 yrs old	typical	7.555	USA	De novo
Sano et al. [34]	1987	45,X/46,XX	no	5 yrs old	typical	4.130	Japan	Familiar
Satre et al. [35]	2004	45,X/46,XX	-	Prenatal diagnosis	-	-	France	Familiar
Ferrier et al. [26]	1965	45,X/46,XX	yes	6 yrs old	typical	R49.000	Switzerland	De novo
Bartolini et al. [36]	1986	45,X/46,XX/47,XXX	-	4 yrs old	typical	Elevated CPK	Brazil	Familiar

## Data Availability

All results are reported within the text.
